# Outcomes of a Short-Duration, Large-Scale Canine Rabies Vaccination Campaign in Central Cambodia

**DOI:** 10.3390/ani14182654

**Published:** 2024-09-12

**Authors:** Keiichiro Tazawa, Amy N. Lewis, Frederic Lohr, Andrew D. Gibson, Martina Mayr, Bengthay Tep, Morany Heng, Stella Mazeri, Waraphon Phimpraphai, Luke Gamble

**Affiliations:** 1 Mission Rabies, Worldwide Veterinary Service, 4 Castle Street, Cranborne BH21 5PZ, Dorset, UK; 2 Faculty of Veterinary Medicine, Kasetsart University, Nakhon Pathom 73140, Thailand; fvetwrp@ku.ac.th; 3 Animal Rescue Cambodia, St. 41BT, House #269, Khan Boeung Tumpun, Phnom Penh 120606, Cambodia; 4 Department of Animal Health and Veterinary Public Health, General Directorate of Animal Health and Production, Khan Meanchey, Phnom Penh 120603, Cambodia; bengthay@gmail.com (B.T.);; 5 Royal (Dick) School of Veterinary Studies and the Roslin Institute, University of Edinburgh, Easter Bush Campus, Midlothian EH25 9RG, Scotland, UK

**Keywords:** rabies, mass dog vaccination campaign, Cambodia, one health, door-to-door

## Abstract

**Simple Summary:**

To advance the national rabies control effort in Cambodia, a coalition comprising government and non-government organizations spearheaded a short-duration canine rabies vaccination campaign in the capital province of Cambodia, Phnom Penh, and the neighboring Kandal Province. The campaign was conducted over 10 days to establish significant herd immunity to stop dog-mediated rabies transmission. The campaign deployed 120 vaccination teams, each consisting of two vaccinators and one tuk-tuk driver. Parenteral rabies vaccines were delivered using a door-to-door method. Utilizing the mobile application, the teams received spatial guidance during the campaign and performed data collection. The campaign vaccinated 74,983 dogs in just 10 days, achieving the district mean coverage of 70.7% among the owned dog population in Phnom Penh Province. The implementation of such a large-scale campaign in a short space of time created a blueprint for mass dog vaccination in urban areas, contributing to upscaling rabies control strategies across Cambodia.

**Abstract:**

Background: WHO and WOAH advocate for annual high-coverage canine rabies vaccination campaigns as the most sustainable approach to eliminate the risk of dog rabies transmission to humans. It is estimated that Cambodia has one of the highest human rabies deaths per capita of any country (5.8 human deaths per 100,000 people), highlighting the urgent need to implement an effective canine rabies vaccination program. To this end, a coalition of government and non-government organizations conducted a pioneering short-duration dog rabies vaccination campaign over 10 days across Phnom Penh and Kandal Provinces in May 2023. Methods: Over 10 working days, 120 vaccination teams, each consisting of two vaccinators and one tuk-tuk driver, traveled door-to-door to deliver parenteral rabies vaccines to all dogs that could be held by the teams or members of the community. Spatial team management and data collection were conducted through the WVS Data Collection Application. Results: A total of 74,983 dogs were vaccinated, giving a mean vaccination rate of 62.5 dogs per team per day. An additional 2145 cats were vaccinated. Of all dogs encountered by the teams, 84.0% could be vaccinated, with 99.1% of those being identified as owned. Post-vaccination surveys of 4356 households estimated a mean vaccination coverage of 70.7% in owned dogs across the districts of Phnom Penh Province. Conclusion: Short-duration, large-scale canine rabies vaccination campaigns can achieve high vaccination coverage using a door-to-door approach in urban centers of Cambodia. Data gathered through the campaign yielded insights to support the refinement and planning of a wider rabies control strategy and is anticipated to drive further support for subsequent campaigns in Cambodia.

## 1. Introduction

Rabies is endemic in many countries across the world, causing approximately 59,000 human deaths annually [1]. Notably, according to the World Health Organization (WHO), dogs are responsible for 99% of these rabies deaths. In order to create the required global momentum to eliminate this disease, a global initiative, United Against Rabies (UAR), was formed by WHO, the Food and Agriculture Organization of the United Nations (FAO), the World Organisation for Animal Health (WOAH), and the Global Alliance for Rabies Control (GARC). UAR has set the global collaborative goal of “Zero by 30”, to create the momentum to eliminate human rabies deaths by canine-mediated rabies by the year 2030 [2]. This initiative advocates the necessity of employing a comprehensive approach to rabies elimination, which includes mass dog vaccination (MDV) campaigns, provision of post-exposure prophylaxis (PEP), and implementation of rabies surveillance. MDV has been demonstrated to be a highly cost-effective public health intervention in multiple endemic settings through the elimination of rabies from the reservoir dog population [3,4,5,6]. While an annual campaign target of 70% vaccination coverage is typically advocated for, elimination has been reported at coverages of 50–70% and is likely influenced by local ecological factors [5,6]. The largest example of successful dog rabies elimination through MDV was led by the Pan American Health Organization in Latin America, in which over 50 million dogs were vaccinated annually at its peak in the 1990s [7,8]. Rabies elimination through MDV has been reported at the city and state levels in N’Djamena, Chad, and Goa, India, respectively [6,9], although examples of successful scale-up to the national level remain absent across much of Africa and Asia.

National rabies control, or elimination, is dependent on the establishment of efficient, recurring annual MDV campaigns across the population. Numerous MDV campaign strategies have been developed, including static point, door-to-door, and capture-vaccinate-release, or a combination of several methods [10,11]. The optimal strategy is determined by the local dog ownership practices and ecology and socio-cultural factors [3,11,12,13]. In areas where owned dogs predominate within the canine population, door-to-door or static point methods can be employed depending on the socio-spatial factors [10,14,15]. Although the door-to-door method can normally achieve high coverage, the static point method can be more efficient given that the dog-to-human interactions and the community awareness are well understood [15]. In addition, capture-vaccinate-release (CVR) teams were deployed in areas with a large free-roaming dog population, but the method requires skilled labor and is a more expensive approach [16]. Finally, Oral Rabies Vaccination (ORV) is emerging as a complementary strategy, especially when free-roaming dog populations are a significant focus [17,18,19,20]. However, challenges remain in implementing ORV, including local infrastructural capabilities to ensure the adequate environment for storage and the need for optimized distribution systems to reach inaccessible dogs [21].

In 2007, Cambodia was estimated to have one of the highest human rabies deaths per capita, with around 5.8 human deaths per 100,000 people [22], yet the true figures are still unknown. Dogs are considered to be the main reservoir species for enzootic rabies virus transmission in Cambodia [23]. At the moment, rabies among wild mammals has not been well documented in Cambodia. The need for improved data collection, heightened public awareness, and targeted prevention measures, namely, the implementation of the surveillance system and organized MDV campaign, was addressed in the National Strategy for Rabies Control and Elimination [24]. However, as of the beginning of 2023, there were not large-scale national MDV activities in Cambodia. The General Directorate of Animal Health and Production (GDAHP) and multiple NGOs, including the Worldwide Veterinary Service (WVS), Animal Rescue Cambodia (ARC), and Phnom Penh Animal Welfare Society (PPAWS), conducted a pilot vaccination project in Phnom Penh Province in September 2019. The one-week MDV campaign vaccinated over 5000 dogs to achieve 52% vaccination coverage in the targeted area, showcasing the feasibility of a larger-scale door-to-door MDV campaign in the province (unpublished data).

With the positive outcomes and garnered support from the local stakeholders from the pilot campaign, a door-to-door MDV campaign was implemented between 22 May 2023 and 2 June 2023 (2023 MDV campaign), targeting the canine population of Phnom Penh Province to fill the current operational gap and create blueprint to scale up the MDV campaigns nationwide. This study describes the methods and outcomes of the first ever province-wide MDV campaign conducted in Cambodia.

## 2. Materials and Methods

### 2.1. Campaign Dates and Location

**Figure 1:**
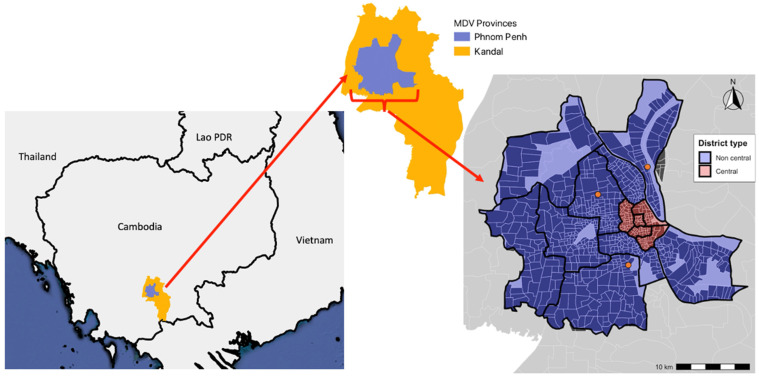
Locations of the provinces included in the MDV campaign and Working Zones and the location of campaign hubs (orange markers) in Phnom Penh Province. Polygons within the district border describe the Working Zones.

The campaign took place on 10 working days between 22 May 2023 and 2 June 2023, with Saturday and Sunday being non-working days. The primary study area was in Phnom Penh Province, which is the central administrative capital of Cambodia. According to the 2019 census, the province had a human population of 2,281,951, with a mean human density of 3361 persons per km^2^ [25]. Phnom Penh Province has a large urban center surrounded by residential areas. As of 2023, there are 14 districts within Phnom Penh Province. Five central districts (Boeng Keng Kang, Chamkar Mon, Doun Penh, Prampir Makara, and Tuol Kouk) have high human population density and high street densities compared to the surrounding suburban districts [26]. Phnom Penh Province was divided into 771 Working Zones, based on the administrative boundaries of its communes (Figure 1) to create regions of a manageable size for vaccination team direction. Campaign hubs were set up in three locations across Phnom Penh Province to aid in campaign coordination. These hub locations were the southern hub (Royal University of Agriculture), the northern hub (Prek Leap National Institute of Agriculture), and the western hub (Phnom Penh Provincial Department of Agriculture, Forestry and Fisheries (PDAFF) office). These hubs played a central role in campaign logistics, providing a place for vaccination teams to gather and the storage for vaccines and equipment. Team Leaders were based in these hubs, coordinating the logistical arrangements for the vaccination teams. The selection of these hubs was based not only on the location but also on the availability of reliable infrastructure. Hence, the universities and the government facilities were preferred. Vaccinations also took place in some districts of Kandal Province bordering Phnom Penh Province. In Kandal Province, village chiefs guided the vaccination teams, and therefore Working Zones were not used. 

### 2.2. The 2023 Campaign Project Approval

The 2023 MDV campaign was approved through a letter of agreement between GDAHP, MAFF, WVS, and ARC. Project implementation approval was obtained from the Phnom Penh Provincial Administration as well as the local district offices. Approval was also given by the Kandal Provincial Department of Agriculture, Forestry, and Fisheries (PDAFF) to expand vaccination projects into Kandal Province. GDAHP and WVS had formal collaboration agreements with the Royal University of Agriculture (RUA) and Prek Leap National Institute of Agriculture (PLNIA), for the use of facilities and recruitment of veterinary students as volunteer vaccinators. All vaccinators and animal handlers who took part in the campaign completed human rabies pre-exposure prophylactic (PrEP) vaccinations. Using the dog population estimated by WVS in 2019 using data from pilot vaccination campaigns (unpublished data), 121,500 doses of vaccine were donated by MSD Animal Health to achieve sufficient coverage.

### 2.3. Pre-Campaign Community Awareness

Community sensitization aimed to provide basic information about rabies prevention and garner engagement ahead of the campaign. Both direct and indirect sensitization methods were used to engage the community. Direct sensitization was carried out by Education Officers who disseminated campaign materials and discussed rabies with the community at key locations, including pagodas, markets, and community centers, four weeks before the campaign. Details of the campaign were also broadcast through loudspeaker announcements and household visits 5-days and 1-day prior to the vaccination teams’ arrival as a result of collaboration with local administrative officials. Radio advertisements were played on three major radio stations, encouraging the community to present their dogs to the door-to-door vaccination teams for free rabies vaccines. Finally, online advertisements on Facebook commenced eight weeks before the campaign and linked to a campaign website providing the latest details of the campaign schedule. The reach of the pre-campaign community sensitization was evaluated by asking dog owners if they were aware of the campaign prior to the vaccination teams’ visit and, if so, how they heard about it.

### 2.4. Campaign Coordination

A total of 120 vaccination teams were deployed, each consisting of two vaccinators and one tuk-tuk driver traveling by tuk-tuk. Vaccination teams were grouped into squads to aid coordination and management. One squad consisted of twelve vaccination teams, giving a total of ten vaccination squads for the campaign. Each squad was coordinated by three to four squad managers who provided both remote and in-field guidance. 

These vaccination teams were assigned to campaign hubs as follows: 60 teams in the southern hub, 24 teams in the northern hub, and 36 teams in the western hub. All vaccinators took part in a one-day training before the campaign on the vaccination method and campaign coordination protocols. The vaccination teams were equipped with the necessary materials for vaccination, in addition to catching nets and dog leads, restraint aids where necessary, and first aid kits. Smartphones with data-loaded SIM cards were provided for the use of the WVS Data Collection Application [27,28] and for communication between teams. 

The Working Zones, described previously, were used to direct the systematic spatial movement of the vaccination teams through the province, with the aim of increasing the homogeneity of vaccination [16,28]. The door-to-door approach to vaccine distribution was used due to its effectiveness, as studies have shown that a high proportion of dogs are owned in Cambodia [22,29] and the majority of bite exposures are caused by owned dogs in Phnom Penh [30]. Two vaccination teams were assigned per Working Zone; however, they worked independently for the majority of the time and combined to form a larger group when additional assistance was required by either team. Squad managers assigned Working Zones to vaccination teams through the WVS Data Collection App, which displayed as polygons on maps within the app. Vaccination teams navigated through their assigned Working Zones between 8 a.m. and 4 p.m. on a working day, with a one-hour break for lunch. Where feasible, an additional Khmer-speaking volunteer was deployed to walk ahead of the vaccination teams to announce their arrival to the local community and request dog owners to fetch their dogs for vaccination.

### 2.5. Vaccination Protocol

Upon encountering a dog, vaccination teams first looked to identify an owner. Where consent could be obtained from an owner, the dog was restrained either by the owner or vaccinators, based on the owner’s availability, the animal’s temperament, and safety. A 1 mL dose of rabies vaccine (Nobivac Rabies^®^—MSD Animal Health) was administered subcutaneously or intramuscularly, according to the manufacturer’s instructions. Vaccinated dogs were marked with animal-friendly paint on the head, enabling short-term identification of vaccination status. Puppies under 3 months of age were also vaccinated. However, there was no revaccination scheduled. Details of every dog encountered, including those not vaccinated, were recorded in the WVS Data Collection App based on direct observation of the dog and through discussion with the owner. Data for each dog included sex, age, the lactation status of females, health status, ownership, and the single most suitable reason for non-vaccination if applicable. The GPS location at the time of data entry was automatically recorded.

### 2.6. Post-Vaccination Survey

Post-vaccination surveys took place in Phnom Penh Province, both during and one week following the end of the vaccination campaign. Seasonal rains precluded the reliability of the paint marks to identify vaccination status during dog sight surveys; however, vaccination coverage could be estimated using household surveys of dog owners due to the high proportion of dogs in the population being owned. A post-vaccination survey took place according to the Working Zones. Each Working Zones was assigned numbers, and a random number generator was used to select for the survey. No specific sample size was defined within each Working Zone due to highly variable settlement types. There were a total of 10 surveyors, who were not part of the vaccination teams. Each surveyor was assigned to a district and was responsible for surveying households there, except for the one assigned to the five central districts. The time interval between the completion of the Working Zones by the vaccination teams and the time of the household survey varied from one to ten days. Surveyors traveled through the allocated Working Zones by motorbike, conducting household surveys between 8 a.m. and 5 p.m. on weekdays. Upon arrival at a household, surveyors sought consent from an appropriate household representative over the age of 18 before commencing the household interview. Enquiries where an appropriate household representative was not present or declined to participate were recorded. At consenting households, the household survey form consisted of several sections, including general household information, dog ownership status, and the vaccination status of all dogs within the household. 

### 2.7. Data Analysis

All data collected for vaccinations and post-vaccination surveys were entered into the WVS Data Collection App and exported in CSV file format. Data were analyzed using R version 3.6.2 [31]. Vaccination coverage was estimated using the post-vaccination survey results by calculating the proportion of dogs that were vaccinated against rabies within three weeks of the survey. The 95% confidence interval (CI) was calculated using the binom.test function [31].

## 3. Results

### 3.1. Campaign Summary

**Figure 2:**
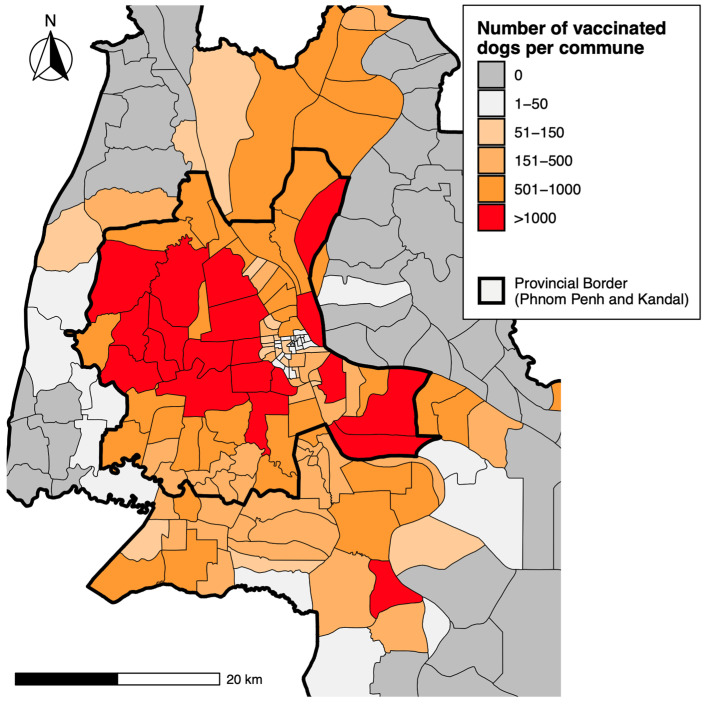
Map of Phnom Penh and Kandal Provinces showing the number of dogs vaccinated per commune.

The vaccination teams encountered a total of 89,285 dogs during the 10 working days, 74,983 (84.0%) of which were vaccinated against rabies. Owners reported that 1795 dogs (2.4%) had been vaccinated recently (no time specified). Of the 74,983 doses of vaccine administered to dogs, 55,578 (76.2%) were delivered in Phnom Penh Province, and 19,405 were delivered in Kandal Province. Figure 2 shows the distribution of dog vaccines at the commune level. An additional 2145 cats were also vaccinated during the campaign, meaning that the total number of rabies vaccine doses delivered to dogs and cats was 77,128.

### 3.2. Campaign Progress

**Figure 3:**
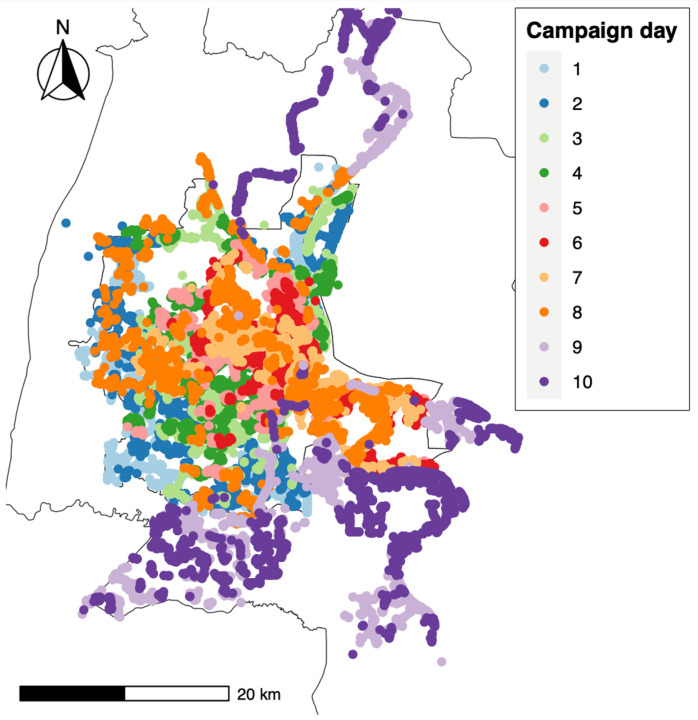
Map of campaign region with points representing the GPS location of dog vaccinations colored by the day of vaccination to show the daily geographic progression of the vaccination workforce.

The daily number of dog vaccinations varied from 4702 (day 1) to 9548 dogs (day 9). The mean dog vaccinations per team per day across the campaign was 62.5 (range: 39.1 on Day 1–79.6 on Day 9). Figure 3 describes the progress of the vaccination teams per campaign day. The vaccination teams began working from the edge of the province, moving towards the central part of Phnom Penh as the project progressed. By day 6, some of the vaccination teams had reached the central part of Phnom Penh Province. On day 8, receiving requests from village chiefs reporting unvaccinated dogs, some vaccination teams revisited areas to increase vaccination coverage. The last two days of the campaign were spent in the districts of Kandal Province, which surround Phnom Penh Province.

### 3.3. Encountered and Vaccinated Dog Demography

Table S1 shows a summary of the demographic details of the 89,286 dogs encountered during the campaign. The sex distribution was skewed towards male dogs, which accounted for 66.6% of the sighted population. Of all the female dogs, 3.0% of them were showing signs of lactation. Puppies under the age of three months represented 15.3% of dogs sighted, and an additional 20% were under one year of age. An owner was identified for 98.2% of dogs encountered, and the remaining 1.8% were considered ownerless. Dogs without an identifiable owner were more challenging to vaccinate, with just 41.6% of the ownerless dogs being successfully vaccinated. Of all dogs encountered, 94.9% were recorded as being healthy, with 86.7% of these able to be vaccinated.

### 3.4. Reasons for Not Vaccinating Encountered Dogs

The vaccination teams could not vaccinate a total of 14,230 dogs encountered during the campaign, representing 16.3% of all dogs sighted. Table 1 shows the reasons for non-vaccination among all dogs encountered, differentiating between dogs older than three months and puppies younger than three months. The predominant reason for non-vaccination was difficulty in catching or handling the dogs, which was the case in 6.1% of all dogs encountered. The second most common reason for non-vaccination was because the dog’s owner was not available during the vaccination team’s visit (3.9% of all dogs). Owners refusing vaccination because their dog had already been vaccinated prior to the campaign represented 2.8% of all dogs encountered. The data for puppies generally shows a lower incidence of owner refusal across all reasons compared to adult dogs.

### 3.5. The Outcome of the Pre-Campaign Sensitization

During vaccination, the vaccination teams asked 20,553 dog owners in Phnom Penh Province about their prior knowledge of the campaign (Table 2). Owners were allowed to provide multiple responses as to the medium; they came to know about the campaign. Of these owners, 61.7% (n = 12,686) reported that they had heard about the campaign via direct or indirect sensitization prior to the arrival of the vaccination teams. Our data shows that the community was better sensitized for the MDV campaign through interactions with village chiefs, family, friends, project education officers, or witnessing local announcement vehicles. Campaign information disseminated through social media such as Facebook or Telegram, and radio did not appear as effective. 

### 3.6. Post-Vaccination Survey Results 

Post-vaccination surveys took place between 31 May 2023 and 9 June 2023, and a total of 6704 households in Phnom Penh Province were surveyed. Of these, 4373 households consented to participate in the survey (65.2%), representing dwellings with a human population of 19,367 people (average 4.43 humans/household). Of the households that took part in the survey, 46.8% (2047) owned at least one dog. The average number of dogs owned in dog-owning households was 2.39 (range: 1–35), and the total number of dogs owned in surveyed households was 4892 dogs. The human-to-dog ratio (HDR) in all participating households in the survey was 3.96. A total of 69.0% (95% CI: 67.7–70.3%) of the dogs recorded were vaccinated against rabies. The vaccination coverage of the central districts and the other 9 districts is described in Table 3. The mean district-level vaccination coverage was 70.7%, with the range between 52.6% and 84.6% (Table 3). The vaccination coverage for owned dogs older than 3 months was 67.4%, whereas for puppies (3 months old or younger), it was 69.2% (95% CI: 65.0–73.0%).

## 4. Discussion

This study describes the first province-wide, short-duration MDV campaign in Cambodia as of June 2023. The findings reported here not only provide operational insights about vaccination rates and methodology to inform the broader national rabies control strategy but also demonstrate the value of such initiatives in understanding dog population demography through the use of a smartphone data collection app. As the global health community advocates for the elimination of dog-transmitted human rabies by 2030, there is a need to rapidly evaluate the suitability of existing vaccination approaches at scale and across diverse geographies to drive innovation and solve remaining barriers to widespread mass dog vaccination. The implementation of this campaign has also established a proof-of-concept that the national scalability is achievable, which aligns Cambodia with international initiatives, such as “Zero-by-30” by UAR or the PEP roll-out by GAVI, the vaccine alliance. 

Campaign duration is of central importance in the structure of any mass vaccination intervention. This MDV campaign was delivered only in 10 working days while achieving substantial herd immunity among the owned dog population. Short-duration MDV campaigns have many benefits, which were leveraged during the successful elimination of dog rabies in Latin America [7]; however, there are limited examples of short-duration MDV initiatives in Asia. In Southeast Asia, the large-scale MDV campaigns have been implemented over a longer period of time. The largest MDV campaign was documented in Bali, Indonesia, where 249,729 dogs were vaccinated through door-to-door and CVR methods to achieve 70% vaccination coverage over a 6-month period in 2010–2011 [32]. The largest door-to-door MDV campaign was delivered in Flores Island, Indonesia, in 2007, vaccinating 172,763 dogs throughout the year, reaching 53% vaccination coverage [33]. The benefits of short-duration campaigns include access to large temporary workforces in the form of volunteers or seconded government staff, as well as the potential to achieve massive public and political engagement through a concentrated awareness campaign [13,34]. The current campaign demonstrates that a large-scale, short-duration vaccination campaign was able to achieve significant vaccination coverage among the owned dog population in Phnom Penh Province through the door-to-door method in the densely populated urban setting of Phnom Penh. The use of mobile technology aided the simultaneous coordination of the large, dispersed workforce of over 120 vaccination teams while rapidly aggregating data in near-real time to inform the strategy while the campaign rolled out. The vaccination of just under 75,000 dogs in ten days provides a benchmark from which to optimize campaign methods for community engagement, efficiency, and scale in the future. 

Phnom Penh Province is of particular significance to rabies control in Cambodia in demonstrating effective methods that can then be applied in other parts of the country. It was chosen as the main location of the campaign due to the availability of baseline rabies-related data from the country’s largest PEP center, the Rabies Prevention Center, the IPC, and the ongoing rabies surveillance project. Robust data on dog rabies make it possible to evaluate the impact of MDV interventions and to intensify or refine the program strategy where expected results are not realized [35]. In addition, as the capital city of Cambodia, holding the vaccination campaign in Phnom Penh also maximized the involvement of key stakeholders, developing shared experience of large-scale MDV campaign implementation. Initiation of mass vaccination interventions in urban settings also provides benefits in availability of workforce, infrastructure, and high population density to maximize output in terms of rate of vaccination and number of individuals benefitted [35,36]. A large part of Phnom Penh Province consists of peri-urban areas, which implies an increased dog bite incidence [37]. Therefore, implementation of a large-scale MDV campaign would make sense in terms of maximizing the impact of current interventions in the capital province.

Analysis of campaign data can uncover opportunities to increase efficiency and vaccination through refinement of the campaign structure. The first day of the campaign represented the lowest vaccination output, with less than 5000 dogs vaccinated, and regions vaccinated during this period were also estimated to have the lowest vaccination coverage. Feedback from the team indicated that vaccination teams were still becoming familiar with the campaign workflow during the initial days, and the allocated Working Zones were also farthest from the Campaign Hubs, resulting in long transit times of up to 90 min before vaccination could begin. These inefficiencies during the early days of the campaign could be addressed through enhancing the initial onboarding training in smaller groups and by focusing initially on vaccinating at locations close to the hubs to maximize time on the field and ensure easier access to assistance where needed. 

While it was possible to vaccinate the majority of the dogs encountered (84%), the campaign methods could be further improved through understanding the reasons that some dogs could not be vaccinated. The campaign data revealed challenges in vaccinating dogs that could not be restrained. Restraint of dogs for parenteral vaccination has been recognized as one of the barriers to increased canine vaccination coverage in other campaigns [14,18,38]. Future improvements could include the introduction of dedicated catching-vaccinate-release (CVR) teams and the use of oral rabies vaccines (ORVs). However, the choice and degree of introduction of new strategies must be judiciously weighted against the cost-effectiveness in a resource-limited setting. For instance, the introduction of catching teams will require increased operational costs along with a skilled workforce, as seen in Goa, India [16,20]. Use of ORV can significantly increase vaccination coverage in areas with low dog accessibility [13,18]. Thailand conducted a trial using ORV to vaccinate free-roaming dogs, suggesting its utility in vaccinating dogs, which cannot be held in a Southeast Asian setting [17]. Nonetheless, ORV would only be possible with legal licensing within Cambodia, and cost-effectiveness should be evaluated as ORV is likely to be considerably more expensive per dose as compared to parenteral rabies vaccines. The use of ORV will most effectively occur alongside a parenteral vaccination campaign. Planning of the proportion of ORV and parenteral vaccines can be assessed using tools such as VaxPlan [13]. 

Furthermore, recruiting a workforce from the local government structure could improve the outcomes and sustainability of the initiatives. In Cambodia, the village animal healthcare workers (VAHWs) framework was created. The VAHWs are expected to play vital roles in providing animal health services and are to be available in every village in all provinces across Cambodia, under the local jurisdictions [39,40]. Their roles in disease surveillance and vaccination programs against foot-and-mouth disease in Cambodia were described in previous studies [39,40]. Additionally, considering further involvement of village-level leadership could be considered, although village chiefs played a role in disseminating the information about the campaign in this initiative. A campaign in Tanzania reported the village-level leadership to engage in the distribution, management, and delivery of the vaccines [41]. A similar campaign model is worth trying in a Cambodian setting as well. 

Public health interventions such as MDV campaigns require sensitization of the community, taking account of the local cultural norms and community structure [42]. The door-to-door vaccination method relies on participation from the dog owners to present their dogs for vaccination when vaccinators come to their home. Therefore, informing dog owners of the campaign ahead of time was vital to success. However, in this campaign, the absence of the dog owners was highlighted as one of the obstacles to vaccinating dogs. We could improve the availability of dog owners in multiple ways. Informing more exact timing of the vaccination teams’ arrival would be a key to achieving this. This will require changing the strategy of the pre-campaign sensitization program as well as the vaccination campaign. According to our results, nearly 40% of the dog owners in Phnom Penh were not aware of the MDV campaign, although we utilized multiple platforms for information dissemination. For the future iteration, it is possible to allocate more time and funding to the direct sensitization methods such as announcement vehicles, village chief visits, Education Officer activities, or increasing visibility of the campaign materials. Other sensitization methods, such as Facebook or radio, did not appear to be the medium from which dog owners obtained the campaign information. As such, these findings can help refine the methods to disseminate the campaign-related information for future iterations, which also impacts the financial allocation within the pre-campaign sensitization program. In addition, we have identified that dogs that could not be held accounted for the highest proportion of non-vaccination. This marks the necessity to deploy skilled handlers who can facilitate hard-to-hold dogs. However, the availability of and cost of such a skilled workforce must be taken into consideration. In the presence of the national MDV program that runs MDV campaigns all year, it would be beneficial to gather a team of skilled handlers. Otherwise, it would not be cost-effective to maintain the workforce. It is also possible to expand on the dog husbandry methods in the pre-campaign training to improve the technique and confidence among the vaccination teams. As for refining the campaign strategy itself, introducing static points for owners to reach out to the vaccination teams themselves would be an option. The introduction of static points at strategically chosen locations in light of the distance from households was proven effective in an MDV campaign conducted in Malawi and Uganda [15,43]. This would also improve cost-effectiveness of the campaign. However, the evidence of the effectiveness of static points in Southeast Asian settings is still scarce. Implementation of the MDV campaign over the weekend could increase vaccination coverage by increasing the availability of dog owners who are at work on weekdays. 

A post-vaccination survey forms an essential part of the MDV campaign assessment by allowing the estimation of vaccination coverage and facilitating data-driven decision-making. The vaccination coverage informs policymakers of the immediate outcomes of the campaign. Our survey showed that the campaign achieved substantial herd immunity in the owned dog population in Phnom Penh Province. The herd immunity was achieved through leveraging mobile technology to ensure comprehensive geographic coverage, as demonstrated in other studies [10,27,28]. The technology-guided approach enables recruiting a workforce who are not familiar with the local geography in the short-duration MDV initiative such as the present campaign. However, it is important to note that our survey results do not cover the whole dog population in Phnom Penh, as the sight-resight surveys could not be conducted. Although the initial plan for the post-vaccination survey was to carry out sight-resight surveys alongside the household surveys, the stock marker used quickly became faint as the dogs got wet during the seasonal rain. Post-vaccination surveyors also reported that the paint marks were removed by some owners. Consequently, the sight-resight survey was discontinued after a few days due to inaccurate data collection. Nonetheless, as most dogs encountered by the vaccination teams were recorded as owned (98.2%), household surveys alone could be a reliable way to evaluate the campaign outcomes. However, it is important to acknowledge the limitations of the household surveys, especially their inability to estimate vaccination coverage in the ownerless dog population. In future MDV campaigns, it would be beneficial to inform owners of the purpose of the paint marks upon vaccination. Alternatively, it is possible to schedule the campaign at a different time of the year with less precipitation. For instance, November could be a more suitable timing than May or June in that the temperature is lower and the precipitation is less than May–June.

Finally, this campaign generated an unprecedented amount of data into the demographic composition of the dog population in central Cambodia. These data provide insights into the dog ecology and ownership practice. The data showed a male-skewed population, which can be compared with other research studying free-roaming dog populations in the region [29,44,45]. The male-skewed characteristic in the Phnom Penh and Kandal dog populations could be explained by the preferential selection for male dogs and a higher mortality rate for female dogs, as seen in other studies [46,47,48]. Additionally, the post-vaccination survey results produced an estimated human-to-dog ratio (HDR) in Phnom Penh Province. The HDR is an indicator to estimate the size of the dog population [49]. The HDR estimated from the post-vaccination surveys in this study provides a rough indicator of the owned dog population size in Phnom Penh in relation to the human population (3.96:1). This figure has changed since the survey conducted in 2009 (4.8:1) [50], indicating there would be a larger dog population in Phnom Penh in 2023. This could be attributed to the rapid urbanization of Phnom Penh city, characterized by an increase in the human population and improved infrastructure [51]. The human population is found to be highly associated with that of dogs, as shown in several studies [52,53]. Although our data did not reveal the exact size of the free-roaming dog population, it is probable that the population of free-roaming dogs has also increased, necessitating a strengthened rabies control strategy. The role of free-roaming dogs in rabies transmission is considered significant, as shown in previous studies [54,55]. Nonetheless, it is important to note that the HDR calculated from our results is not reflective of the actual dog population, as surveyors were not explicitly instructed to survey households regardless of dog ownership, indicating there is a sample selection bias.

Looking at the results of the MDV campaign from multidisciplinary perspectives would be of paramount importance. For example, investigating the MDV campaigns from a financial point of view provides an opportunity to evaluate the sustainability and cost-effectiveness of the campaign. However, due to the scale of the campaign and the pending financial disclosure, we were unable to ascertain the precise cost per vaccinated dog. This gap underscores a crucial area for future research. It is imperative that subsequent efforts be directed towards conducting a comprehensive cost analysis post-financial disclosure. 

## 5. Conclusions

This report on the first ever provincial-wide, large-scale MDC campaign in Phnom Penh and Kandal Provinces in 2023 has outlined the preliminary but significant outcomes of the campaign. The campaign has made considerable progress in vaccinating a large segment of the canine population in Phnom Penh Province in a short space of time using a door-to-door approach. The data collected throughout the campaign will serve as a valuable baseline for refining campaign strategy for subsequent iterations and for other districts within Cambodia. The findings underscore aspects that require further improvements, such as the vaccination strategy and preparation, including training and pre-campaign sensitization, to augment the campaign outcomes, developing a more tailored approach. Future analysis on the cost-effectiveness of the campaign would be useful to evaluate the sustainability of this present campaign strategy. Overall, the 2023 mass canine rabies vaccination campaign laid the groundwork for effective and efficient rabies control efforts in Cambodia and similar resource-limited settings.

## Figures and Tables

**Table 1:** Summary of the reasons given for not vaccinating encountered dogs.

Reasons for Non-Vaccination	# Among Dogs Older Than 3 Month Old (%) (n = 75,596)	# Among Puppies under 3 Months Old (%) (n = 13,690)	# Among Dogs (%) (n = 89,285)
Dog could not be held	5243 (6.9)	160 (1.2)	5403 (6.1)
Owner was not present	3365 (4.5)	150 (1.1)	3515 (3.9)
Refused by the owner due to recent vaccination	2367 (3.1)	111 (0.8)	2478 (2.8)
Refused by the owner as the owner had no time to engage	382 (0.5)	14 (0.1)	396 (0.4)
Refused by the owner due to a lack of trust in vaccination teams	805 (1.1)	75 (0.5)	880 (1)
Refused by the owner due to a lack of awareness	382 (0.5)	16 (0.1)	398 (0.4)
Other reasons	1002 (1.3)	158 (1.2)	1160 (1.3)
Total	13,546 (17.9)	684 (5)	14,230 (15.9)

**Table 2:** Distribution of sensitization channels for pre-campaign sensitization.

Types of Sensitization	Total
Saw/heard the campaign advertisement	12,686 (61.7)
Visited by village chief	2818 (13.7)
Heard from family or friends	2645 (12.9)
Heard the local announcement vehicle	2607 (12.7)
Met Education Officers	2473 (12.0)
Saw campaign materials	2430 (11.8)
Facebook	1548 (6.8)
Telegram	310 (1.5)
Radio	377 (1.6)
Did not see/hear the campaign advertisement	7867 (38.3)

**Table 3:** Estimated vaccination coverage for districts in Phnom Penh Province (central districts represent Boeng Keng Kang, Chamkar Mon, Doun Penh, Prampir Makara, and Tuol Kouk).

Districts	Number of Dogs Surveyed	Number of Surveyed Dogs That Were Vaccinated	Vaccination Coverage in % (95% CI)
Central districts	99	67	67.7 (57.5–76.7)
Chbar Ampov	555	364	65.6 (61.5–69.5)
Chraoy Chongvar	459	325	70.8 (66.4–74.9)
Dangkao	849	725	85.4 (82.8–87.7)
Kamboul	984	518	52.6 (49.5–55.8)
Mean Chey	173	101	58.4 (50.7–65.8)
Praek Pnov	235	188	80 (74.3–84.9)
Pur SenChey	928	597	64.3 (61.2–67.4)
Russey Keo	123	104	84.6 (76.9–90.4)
Saensokh	379	295	77.8 (73.3–81.9)
District average			70.7

## Data Availability

Data is contained within the article or Supplementary Material.

## Supplementary Materials

The following supporting information can be downloaded at: https://www.mdpi.com/article/10.3390/ani14182654/s1, Table S1: Table of the demographics of all encountered dogs disaggregated by vaccination status.
