# Supplementary material for: Outcomes of a Short-Duration, Large-Scale Canine Rabies Vaccination Campaign in Central Cambodia
Source: Animals (Basel). 2024 Sep 12;14(18):2654. doi: 10.3390/ani14182654 (PMC11429455; doi:10.3390/ani14182654)
Supplement: Supplementary file 1 [file animals-14-02654-s001.zip › animals-3104878-supplementary.pdf]

Table S1. Table of the demographics of all encountered dogs disaggregated by vaccination status.

	**Vaccinated Dogs (n = 74983)**		**Unvaccinated Dogs (n = 14302)**		**Total encountered dogs**	
	Number	% within category	Number	% within category	Number	% of all encountered dogs
**Sex**						
**Male**	46653	86.7	7142	13.3	**53795**	**60.3**
**Female (Non-lactating)**	24392	89.9	2739	10.1	**27131**	**30.4**
**Female (Lactating)**	2389	87.9	329	12.1	**2718**	**3.0**
**Unknown sex**	1549	27.5	4092	72.5	**5641**	**6.3**
**Age**						
**Puppy (< 3 months old)**	13001	95.0	689	5.0	**13690**	**15.3**
**Juvenile (> 3 months, < 12 months old)**	15726	88.0	2148	12.0	**17874**	**20.0**
**Adult (> 1 year, < 5 years)**	35727	78.9	9538	21.1	**45265**	**50.7**
**Old (> 5 years old)**	10529	84.5	1927	15.5	**12456**	**14.0**
**Ownership**						
**Owned**	74303	84.8	13349	15.2	**87652**	**98.2**
**Not owned**	680	41.6	953	6.7	**1633**	**1.8**
**Health Status**						
**Heathy**	73466	86.7	11284	13.3	**84750**	**94.9**
**Not healthy**	1517	33.5	3018	66.5	**4535**	**5.1**
